# Resilience and active inference

**DOI:** 10.3389/fpsyg.2022.1059117

**Published:** 2022-12-22

**Authors:** Mark Miller, Mahault Albarracin, Riddhi J. Pitliya, Alex Kiefer, Jonas Mago, Claire Gorman, Karl J. Friston, Maxwell J. D. Ramstead

**Affiliations:** ^1^Center for Consciousness and Contemplative Studies, Monash University, Melbourne, VIC, Australia; ^2^VERSES Research Lab, Los Angeles, CA, United States; ^3^Department of Computing, Université du Québec à Montréal, Montreal, QC, Canada; ^4^Department of Experimental Psychology, University of Oxford, Oxford, United Kingdom; ^5^Department of Philosophy, Monash University, Melbourne, VIC, Australia; ^6^Integrated Program in Neuroscience, Department of Neuroscience, McGill University, Montreal, QC, Canada; ^7^Division of Social and Transcultural Psychiatry, McGill University, Montreal, QC, Canada; ^8^Wellcome Centre for Human Neuroimaging, University College London, London, United Kingdom; ^9^MIT Senseable City Lab, Massachusetts Institute of Technology, Cambridge, MA, United States

**Keywords:** active inference, resilience, elasticity, plasticity, robustness, complex adaptive system, inertia

## Abstract

In this article, we aim to conceptualize and formalize the construct of resilience using the tools of active inference, a new physics-based modeling approach apt for the description and analysis of complex adaptive systems. We intend this as a first step toward a computational model of resilient systems. We begin by offering a conceptual analysis of resilience, to clarify its meaning, as established in the literature. We examine an orthogonal, threefold distinction between meanings of the word “resilience”: (i) inertia, or the ability to resist change (ii) elasticity, or the ability to bounce back from a perturbation, and (iii) plasticity, or the ability to flexibly expand the repertoire of adaptive states. We then situate all three senses of resilience within active inference. We map resilience as inertia onto high precision beliefs, resilience as elasticity onto relaxation back to characteristic (i.e., attracting) states, and resilience as plasticity onto functional redundancy and structural degeneracy.

## 1 Introduction

Over the last few decades, there has been a multidisciplinary effort to investigate *resilience*. But the word “resilience” is, in practice, polysemous: it is used in several different ways in relevant literature. In this article, we first engage in some conceptual analysis to disentangle three uses of the term, which distinguish three complementary aspects of resilience, conceived of as processes. We then examine resilience from the point of view of active inference, a new physics-based modeling framework for complex adaptive systems. After briefly introducing active inference, we examine how each sense of “resilience” (as inertia, elasticity, and plasticity) can be given a straightforward and formal interpretation within the active inference framework. Explicitly, we map inertia onto high precision beliefs, elasticity onto the ability to seek out characteristic states, and plasticity onto the capacity for functional redundancy and structural degeneracy, defined in a technical sense (Sajid et al., 2020). Rethinking our understanding of resilience in formal terms is important as it allows us to model systems which have resilient properties in relation to their environment. Given a context, we can establish the kinds of patterns which can lead an agent to maintain itself through time. This can be applied to adaptive or maladaptive processes, such that a simulation may be used to reinforce the resilience of a process, or destabilize a maladaptive one.

## 2 Three senses of resilience

In the resilience literature, in addition to disagreements about the locus of resilience (McEwen, 2003; Kirmayer et al., 2009; Masten and Wright, 2010; Ungar, 2011; Dresen et al., 2019), there is also some muddle about what the concept of “resilience” denotes or means (Anthony, 1987; Masten, 2002; Herrman et al., 2011; Reghezza-Zitt et al., 2012; Helfgott, 2015; Woods, 2015; Rose, 2017). We have conducted an analysis of the concept of resilience, based on an extensive literature review (Holling, 1973; Cairns et al., 1977; Westman, 1978, 1986; Cicchetti and Curtis, 2006; Lerner, 2006; Soule, 2006; DuMont et al., 2007; Masten, 2007; Lemery-Chalfant, 2010; Rutter, 2012; Duchek, 2014; Standish et al., 2014; Luthar, 2015; Juster et al., 2016; McJunkin and Rieger, 2017; Santarelli et al., 2017; Cousijn et al., 2018; Dresen et al., 2019; Mertoguno et al., 2019; Den Hartigh and Hill, 2022). We believe, consonant with the analysis in Den Hartigh and Hill (2022), that there are three closely related concepts at play in discussions of resilience, which are not usually distinguished from one another. The word “resilience” is used in the literature to mean either: (i) inertia, i.e., the ability to resist change when subjected to a disturbing force, roughly synonymous with robustness (Westman, 1978; Woods, 2015; Scheffer et al., 2022); (ii) elasticity, i.e., the ability to flexibly return to good states following a perturbation (Cairns et al., 1977; Gapp et al., 2014); and (iii) plasticity, i.e., the ability to expand the repertoire of good states—and courses of action—in the face of a changing environment (Cicchetti and Curtis, 2006; Soule, 2006; Duchek, 2014).

Intuitively, we regard an agent as being resilient when it is able to successfully weather stressful situations, and in particular to return to well-functioning after suffering a setback or insult of some kind.^1^ This is evident in the etymology of the word “resilience”: the Latin *resiliō* means, quite literally, to bounce or spring (*saliō*) back (*re*). The resilient agent is thus one that can, when perturbed, bounce back to a desirable state. Arguably the two other senses of “resilience” we identified in the literature are distinct from, though closely related to, resilience in this original sense: inertia has the characteristic of weathering stress, but not by bouncing back (since the strictly inert object is never moved in the first place); and expanding one’s tolerance to change is more a means of creating resilience (as elasticity) than a subgenre of resilience. Despite these verbal issues however, these concepts are clearly closely interrelated and are all crucial to understanding the processes of resilience. In the remainder of this section, we briefly review these three uses of “resilience.” In the following sections, we leverage the tools of active inference to provide a formalism in which to articulate all three aspects—a formalism that allows us to simulate, estimate, and predict the key facets of resilience quantitatively.

### 2.1 Resilience as inertia (being impervious to change)

First, resilience can mean inertia, or the ability to resist change in structure or function (Holling, 1973). An object with inertia is one that resists displacement by resisting being moved if it is immobile, and resisting changing direction if it is moving along a path. Hard materials like diamond and concrete exhibit resilience as inertia as they are not typically deformed by the forces that act upon them, and instead tend to deform most materials that impact them. Organic systems exhibit resilience as inertia by retaining a physical or functional state at rest or when acting in their environment. For example, a person can stay standing, walking, or running when there is wind—we do not dissipate.

### 2.2 Resilience as elasticity (bouncing back to set points)

Resilience can also mean elasticity (Westman, 1978, 1986; Reghezza-Zitt et al., 2012), which is the disposition and capacity to return to good states after being forced to depart from them due to environmental perturbations. A rubber band is resilient in this sense, as it can stretch away from its normal state, and return to it easily, without tearing.

In living systems, this capacity to return to characteristic states is usually attributed to the functions of homeostasis and allostasis (McEwen, 2003), in which an organism can inhabit different states—which are adaptive in a given context—and eventually return to their characteristic states. Homeostasis refers to the capacity of an adaptive system to maintain those relatively stable internal conditions that are necessary for their survival (Recordati and Bellini, 2004; Herrman et al., 2011; Cummins and Wooden, 2014). For example, plants adapt and change their internal structures, and thus occupy pro-optimal states, in response to an environmental signal (e.g., phototropism, growing in the direction of the sun). In animals, this is done in part through automatic “reflex actions” (e.g., burning fat when we are hypoglycemic, keeping body temperature and blood oxygen saturation levels within viable bounds). Maintaining oneself in a persistent, life-conducive set of states is a central process for all biological organisms. To the degree that an organism can self-regulate in ways that allow it to persist over time in the face of perturbations (from within its own body or from the world), it can be said to be resilient. As such, all persisting self-organizing systems are resilient to some degree.

However, it is not enough to reactively avoid dangerously unexpected states, as captured by homeostasis. Successfully maintaining life-conducive steady states requires that the organism be able to anticipate future disturbances and opportunities and so adjust itself and its chosen courses of actions to optimize future fitness. Hence, in complex systems, resilience requires an ability to successfully *plan*. This online evaluation of possible future needs and selection of courses of action to meet them is known as allostasis (Sterling and Eyer, 1988; Corcoran, 2021). Roughly speaking, if homeostasis is the controlled process of returning to set-points, allostasis is the preemptive control of set-points themselves to meet the demands of a situation. In other words, allostasis is the altering of structure and function of the agent to finesse homeostasis. For example, in addition to burning fat when blood sugar levels drop (a homeostatic process), we mitigate or nuance autonomic responses by indulging in a quick snack (an allostatic process).

### 2.3 Resilience as plasticity (expanding one’s repertoire of good states)

Finally, resilience can mean plasticity or growth, the ability to explore or increase the repertoire of states that are compatible with thriving and a healthy state of being (Duchek, 2014), reducing the probability that a difficult event will dissipate or destroy the agent (Soule, 2006; Dresen et al., 2019). Anthony (1987) described this process as *psycho-immunization*, wherein the agent develops some form of resistance to future risk by learning from previous and current experiences and difficulties, and better equipping the self to handle future risks. Throughout its development, an agent must grasp at the opportunities presented by its ecological niche that maximize the potential for plasticity (Masten and Wright, 2010), increasing the breadth of safe states. Perhaps paradoxically, some lines of research suggest that, at least within certain bounds, exposure to psycho-social stressors and deprivation during the early stages of life can, at least in some people, result in the emergence of beneficial, protective traits, such as secure attachment, educational engagement and achievement, and prosociality, later in life (Gapp et al., 2014; Chaby et al., 2015; Santarelli et al., 2017). This seeming paradox is dissolved by noting that such individuals tend to develop psychological traits, such as hyper-vigilance, that benefit them in a threatening environment. This illustrates how resilience emerges in the dynamic interplay between the agent and its environment. This dynamic interplay leading to learning extends to learning allostasic and homeostatic states. In what follows, we revisit these distinct facets of resilience and cast them as formal aspects of sentient behavior; namely, active inference and learning.

## 3 Active inference

The active inference framework provides us with formal tools well-suited to help us understand resilience, which we take to be the key feature of any complex adaptive system with the ability to persist over time; namely, the tendency to return to characteristic states that enable its continued existence. Using this approach, we can frame the extant senses of resilience, and provide a formal account of resilience that situates—and relates—each of the concepts discussed above. First, we review active inference. Then, in the following section, we examine how each of sense of resilience can be fleshed out in the ensuing frame-work.

### 3.1 Overview of active inference

Active inference is a process theory derived from the variational free energy principle (FEP) in theoretical biology and statistical physics. The FEP is a principle of least surprise (technically, a variational principle of least action or constrained maximum entropy principle): it says that systems that exist, on average, do what is characteristic (i.e., unsurprising) of them, given the kind of thing that they are. This is a tautology, but its consequences are profound. Said differently, in order to persist as a bounded, separable system (and not merely dissipate into the embedding environment), a system that exists must on average return to its characteristic states, which are limited in number. This means that the probability distribution over its states (including the states of its sensory input channels) must have relatively low entropy. From this perspective, agents are fashioned by natural selection, development, and learning to expect to sense the consequences of their continued existence; this is sometimes called self-evidencing (Hohwy, 2016).

The FEP originated as a theory of the function, structure, and dynamics of the brain and can be applied to provide an understanding of any adaptive system that persists over time. When the FEP is applied to sentient (i.e., sense making) systems we get a process theory called active inference. This process theory allows us to model and understand the dynamics of adaptive systems at different scales of self-organization, from the cellular to the societal. The FEP provides a first-principles account of adaptive, belief-driven behavior, by providing a general formalism to model the representational capacities of living tissues (and physical systems more generally) (Friston et al., 2017; Ramstead et al., 2022). One of the central innovations of active inference is the re-conceptualization of living systems generally, such as bodies, brains, and even ecosystems, as machines driven by probabilistic prediction. According to active inference, the dynamics or behavior of the situated brain and body entail an implicit statistical (i.e., generative) model (Ramstead et al., 2020), which enables us to cast the very existence of an agent as a process of making inferences about the causes of incoming sensory perturbation—perturbations that stem both from within the body and from the extra-personal world.

In this setting, the internal states of something come to encode a “best guess” about the state of the world (technically, internal states parameterize a posterior or conditional probability density over external states, which is called a variational density). This best guess is elaborated under a model that generates the sensory consequences of external causes; including the action of the agent in question. Over time, this “world model” comes to instantiate knowledge about the environment’s statistical structure and contingencies. Discrepancies between what the organism predicts, based on its probabilistic beliefs, on the one hand, and the actual sensory feedback that it registers, on the other hand, is a quantity called variational free energy; and the FEP says that things that exist follow paths of least free energy (under Gaussian assumptions, this free energy is simply a prediction error). Free energy is minimized in one of two ways: either by updating the model itself (i.e., perception and learning), which makes our predictions more like the data that we sense; or altering the body and/or world, to make it better fit our predictions (i.e., action and niche construction).

Existence in a changing world requires that one must plan ahead to a greater or lesser extent. Accordingly, adaptive systems must not only consider how well they perform in the present moment, but also how they will fare in the future if they pursue this or that course of action. To account for this temporal depth, agents need to evaluate the free energy that they expect to encounter in the future, contingent on specific courses of action.

Arguably, much of what we take to be central to human intelligence—perception, action, attention, emotion, learning, social interaction, culture—can be modeled within this simple framework of prediction generation and error reduction (Hohwy, 2016, 2018; Veissière et al., 2020; Hesp et al., 2021; Parr et al., 2022). So long as the agent is able to minimize its prediction errors or free energy, it will typically succeed at remaining well-adapted to its environment.

### 3.2 Active inference, free energy, and expected free energy

The phrase “active inference” generally refers to a process theory that describes how the FEP may be instantiated in particular intelligent systems, allowing us to describe the path of least surprisal taken by an agent. In applied computational modeling work, however, its meaning is usually narrower: in such contexts, “active inference” refers to a more specific family of implementations of the general process theory, as inference processes performed under partially observable Markov decision processes (POMDPs). These kinds of generative models are generic but assume the world (and body) can be explained in terms of discrete states—states that can be fine-grained or coarse-grained, depending upon what is apt to minimize free energy or surprise.

There are three main kinds of belief updating in such models. First, there is inference or state estimation, i.e., inferring the state of the world from the data to which one has access; this is taken as a formal model of perception. There is a special kind of state estimation that corresponds to inferring “what I must be doing, given what I know and what I have sensed currently”: this kind of inference over beliefs about possible courses of action is, appropriately enough, called policy selection, which in this case is a form of planning as inference (Attias, 2003; Botvinick and Toussaint, 2012). Second, there is parametric learning or parameter estimation, i.e., learning the value of the parameters of the generative model (e.g., the likelihood matrix and transition matrices). Finally, we have structure learning; namely learning the structure and architecture of generative models *per se* (Gershman and Niv, 2010; Friston et al., 2017).

**FIGURE 1:**
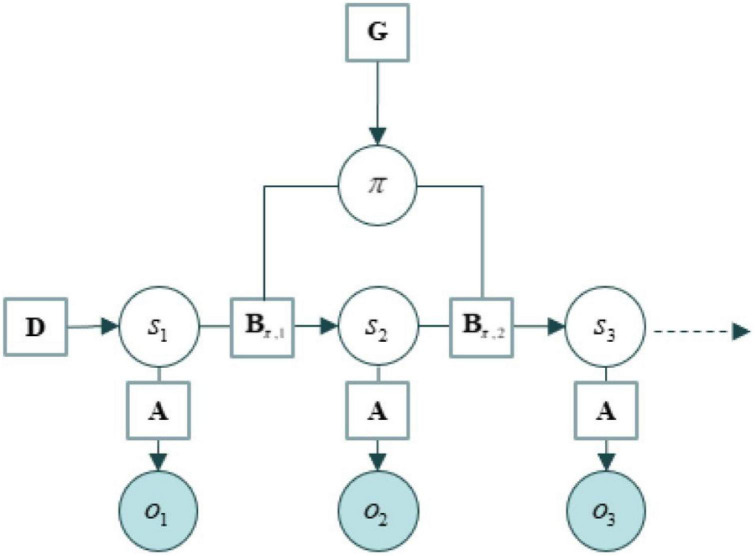
An example of generative model taken from Albarracin et al. (2021).

We use the tools of active inference to formalize the structure of the agent environment system as a generative model (Ramstead et al., 2020, 2022), which contain states, directed edges between states, and parameters associated with those edges. The structure of these models is usually described in an easy-to-remember ABC…fashion, where the *A* corresponds to the likelihood part of a generative model and everything else corresponds to priors over states and their trajectories. See an example of their structure in Figure 1. In brief: As summarized in Table 1.

**TABLE 1:** Parameters used in the general model under the active inference frame-work.

o	Observations or sensory states of an agent
s	Hidden or external states
A	Likelihood matrix that captures beliefs about the mapping from observations to their causes (hidden)
B	Transition matrix that captures beliefs about the mapping between states at one time step to states at the next time step
C	Prior preference matrix that captures the preferred observations for the agent, which will drive their actions
D	Priors that capture beliefs about base rates of occurrence of the hidden states
E	Prior preferences for policies in the absence of data
F	Variational free energy
G	Expected free energy
π	Policy matrix that captures the policies available to an agent
γ	Precision of beliefs

We explain the generative model symbols that refer to different matrices and elements which are connected through them. Albarracin et al., 2022b.

State estimation depends on the likelihood matrix, denoted *A*, which lists the probability of some data under the assumption that the world is in a certain state. *B* matrices encode transition probabilities among states, and therefore the evolution of the world over time. State transitions are dependent on courses of action or policies. The final sensory outcomes—that agents prefer—are specified in a matrix denoted *C*. Similarly, priors over initial states of a new context are encoded in a matrix labeled *D*. In empirical Bayes (i.e., deep or hierarchical generative models), priors over initial states are updated when the context changes. Policy selection is also guided by beliefs that are accumulated over contexts. These play the role of habits: a prior belief about which policies to pursue in this context, in the absence of sensory evidence. These habits are encoded in an *E* matrix. Finally, free energy is denoted *F*, and the expected free energy of plausible policies is encoded in a *G* vector.

Crucially, to each of these parameters and beliefs one can associate a *precision* or inverse variance. Precision is a key construct in data assimilation and computational neuroscience. It basically quantifies confidence invested in a belief or model parameters (e.g., likelihood mappings or prior transition probabilities). For instance, the contribution of expected free energy to policy selection is modulated by the a precision term denoted γ: when it is high, policy selection is driven more by expected free energy than habitual priors (c.f.: model-based vs. model free formulations in reinforcement learning). In other words, the precision of beliefs about policies simply reflects the confidence an agent has in her plans. This, in turn, can be thought of as arbitrating between habitual responses in any given context and deliberative (although usually sub-personal) goal-directed behavior; where the goal is to minimize expected free energy. Since free energy can be thought of as a surrogate for surprise, goals can always be cast as minimizing expected surprise or uncertainty of one sort or another.

The expected free energy of a policy can be rearranged in different ways that reveal the preference and information-seeking aspects of goal-directed behavior. One of the most revealing decompositions is into pragmatic and epistemic value, respectively. Agents that minimize expected free energy are motivated to act within the world according to two core incentives. On the one hand, agents may leverage their probabilistic models of the world and choose their actions to obtain preferred (unsurprising) outcomes. These are called pragmatic or instrumental actions. On the other hand, agents often choose to act in ways that allow them to learn more about the environment and seek out those observations (i.e., surprising or novel ones) that allow them to most efficiently update their models. These are called epistemic actions, which are chosen to maximize information gain.

Under the FEP, instrumental and epistemic imperatives for actions—or affordances—are automatically balanced given beliefs about states of the world and their inherent uncertainty. If agents are unsure about the state of their environment, they will usually select policies that have high epistemic value. On the other hand, if an agent already knows its environment well—or has very precise preferences—then their actions will be guided more strongly by an instrumental drive. Technically, this dual aspect of planning can be framed as Bayes optimal, in the sense of complying with the principles of optimum Bayesian decision and design, respectively (Lindley, 1956; Berger, 2013). The FEP places both of these imperatives on the same footing, and resolves the conflict between exploration and exploitation—in the right order (i.e., agents act to disambiguate their situation, and then seek their preferred outcomes). Therefore, an agent’s behavior is such that the chosen policy has the highest combined instrumental and epistemic value.

### 3.3 Second-order inference and affect

In recent work on active inference (Hesp et al., 2021), affective inference is formalized as a form of second-order inference: these are inference about our inferences, which take posterior state estimations at one level of the generative model and pass them onto a super-ordinate layer, as data requiring further explanation. In this way, active inference agents tune their adaptive behaviors to changes in how the generation of error itself changes over time; specifically, changes relative to expectations about velocity and acceleration of error generation and minimization. As discussed below, these second-order, affective dynamics play a key role in keeping predictive agents poised, in a meta-stable fashion, between well-known and unknown niches. Crucially, optimal inference does not mean avoiding surprising data *per se*. To the contrary, any system that minimizes its expected free energy is also, *ipso facto*, organized to seek out interesting *slopes of error* and has a propensity to actively seek out surprises.

## 4 Resilience from the active inference perspective

Before we describe resilience through the lens of active inference, we motivate the endeavor. Why use active inference to study resilience? One reason is theoretical unification: as we shall see, active inference allows us to provide an account of resilience that does justice to all the main ways in which the concept has been deployed in the specialist literature. A second reason is that it enables us to explain how each of the three processes of resilience that we identified relate to, and complement, each other. Finally, our active inference formulation of resilience also allows us to see that and, especially, *why* inertia and elasticity are not sufficient on their own for a system to be resilient in a robust sense. We will argue that without plasticity, agents may have, as it were, too much resilience for their own good, and may be caught in locally optimal, but globally sub-optimal, solutions.

### 4.1 Inertia as high precision

We now turn to our active inference account of the three concepts or processes of resilience. The first, and perhaps most taken for granted, aspect of resilience is inertia, or robustness, which denotes the fact that an agent has the ability to withstand change, without affecting its internal structure or dynamics. More precisely, inertia entails that an agent’s internal states and parameters do not change given some disturbance.

This is exactly analogous to inertia in physics, which roughly speaking scores how difficult it is to get some system to deviate from its current trajectory (or to get it moving if it is motionless). In the active inference framework, this corresponds quite naturally to *beliefs endowed with high precision* (Kim et al., 2022). With high enough precision over the model, sensory perturbations do not disturb the agent enough to move it away from prior set points. Consider an aforementioned example of a person being able to stay walking, or even running, when there is wind. The physical structure of the person is not threatened by the wind around the person. The precision (in the sense of the inverse of the variance of a distribution describing the person’s state) of the connections between molecules (such as skin cells) that form the person is higher than the degree of disturbance entailed by the environment. This sense of resilience (inertia) is also occurring at a coarser grain, where shorter-term perturbations are not causing disturbance. For instance, internal biological processes in a person, such as breathing and blood circulation results in a variety of different states. But, the physical structure of the person remains intact, unperturbed by the different molecules entering, moving within, and exiting the person.

Another perspective on this is to note that an agent’s internal states and configuration encode beliefs, which means that inertia implies a resistance to belief updating. This can be guaranteed if prior beliefs are very precise and resistant to revision by sensory evidence. Recall that in active inference, sensory evidence is sampled via action. In other words, an agent is in part the author of her own sensations and can therefore sample the world in a way that conforms to her precise beliefs and predictions. A simple example of this would be homeostasis that keeps certain (interoceptive) sensations within very tight bounds; in virtue of precisely held prior beliefs that these “essential variables” should be close to homeostatic setpoints. One can generalize this idea beyond interoception to other forms of niche construction; from keeping the extrapersonal world predictable, clean and tidy; through to cultural niche construction by adhering to precisely held beliefs in social exchanges (Seth and Tsakiris, 2018).

#### 4.1.1 Rigidity

However, with high precision also comes low flexibility. High-precision beliefs act as a strong inductive bias and limit the capacity of an agent to update their prior beliefs and change in the face of changing circumstances. A very rigid agent does not have the ability to explore new options, since it is entrenched in its own beliefs. Such an agent may struggle with volatility. The only way for an agent with overly precise beliefs to maintain itself in its characteristic states is to remain in low volatility environments.

When an agent has a high precision policy that occupies low volatility states, resilience would take form of selectively sampling for states with little unexpected surprise. For example, people who believe that the Earth is flat may still gather new evidence to confirm or disconfirm their beliefs (i.e., engage in epistemic actions). However, if the precision of their prior beliefs is too high, they will not be receptive to evidence that challenges their core assumption that the Earth is flat. See Albarracin et al. (2022a) for a simulation of a system inoculating its high precision beliefs and policies by operating in echo chambers. Such people could be examples of agents that are resilient (in the sense that their beliefs are robust), while still holding a suboptimal model of the world. Within their specific social niche, these people will reduce expected free energy by engaging in such kinds of actions that align with their core assumptions, since they are shared by others (as they are frequenting situations which support and reinforce their beliefs).

### 4.2 Elasticity as relaxation back to characteristic states

Resilience as elasticity refers to a system’s ability to “bounce back” to a previous state after some disturbance. For example, rubber is a resilient material in the sense that it returns to its initial state when deformed by a physical force applied to it: it is elastic. A ceramic mug, in contrast, is inelastic: if its physical structure is perturbed to any significant degree, it shatters and is not able to recover that structure.

We can understand the anticipatory dynamics of biological resilience through the lens of active inference. The elastic aspect of resilience then can be modeled in terms of homeostasis and allostasis—the processes that allows such a self-organizing system to anticipate and compensate for various forms of volatility and uncertainty. In effect, resilience as elasticity (as the capacity to return to a set-point) can be understood as the most basic feature of any active inference agent, which is its capacity to return to its characteristic states. This kind of resilience rests upon planning over extended temporal horizons; namely, deep temporal models that afford a route back to preferred states. In other words, the very distinction between allostasis and homeostasis depends upon the pre-emptive policies that anticipate the consequences of action in the distant future. In this sense, resilience is conferred upon agents whose priors encompass policies with temporal depth, and therefore a future-pointing kind of self-evidencing that can circumnavigate short-term surprises in an unpredictable world. Active inference under deep generative models provides a powerful formal model of homeostasis and allostasis (Corcoran, 2021).

Suppose a person goes out for a run. We know that humans have a high precision expected state of body temperature being around 37^°^C. So, the person would produce sweat as a cooling mechanism for the body while on the run. This is homeostatic elasticity. The person would also take some water in a bottle to the run to ensure enough water in their body to produce more sweat, and maybe draw their curtains close at home before going on the run so they have a cool space to return to after their run. This is allostatic elasticity, with deep temporal models as planning was conducted over extended temporal horizons to bring the person back to preferred states.

In short, the *C* vector provides the agent with homeostatic set-points specified in terms of preferred sensory data. The active inference scheme essentially tells us how agents generate their preferred sensory data through commitment to certain policies, which they infer by minimizing expected free energy.

#### 4.2.1 Bad bootstraps

For complex organisms like us, who are endowed with models that make inferences not just about the present but also about future states (i.e., a deep temporal model), lifelong resilience often requires more than these first two forms of resilience that we have discussed. While these can lead to various forms of local stability, this stability can itself sometimes become sub-optimal, and so undercut longer term, more global patterns of resilience. To be resilient, it is not sufficient that a self-organizing system merely persist through time. The fact is that, even in minimizing free energy, agents can become suboptimal relative to other agents in the same situation—based on important changes in the generative model they entail. However, this persistence may involve adaptation to a changing environment (Constant, 2021). This is crucial for the wider discussion about the importance of resilience—the fact that a system is resilient according to the first and second definitions that we provided (i.e., inertia and elasticity) does not guarantee that the agent is in fact flourishing, and responding responds adaptively to its environment in the long term. At higher levels of organization and temporal scales, the very structure of the generative model—under which active inference unfolds—has itself to be learned. This is known as structure learning in radical constructivism or Bayesian model selection in statistics (and perhaps natural selection) (Gershman and Niv, 2010; Tenenbaum et al., 2011; Gershman and Blei, 2012; Vanchurin et al., 2022). One key aspect of this kind of structure learning is the scope of policies entailed by policy selection. For example, in an unchanging world, if I see myself behaving in a particular way, I will learn that this is the kind of thing I do and develop habits, which could be epistemic habits (e.g., always watching the news at 10 o’clock) (Friston et al., 2016). However, in a changing world these habits may no longer be apt, and I may need to extend the repertoire of explanations for—and explorations of—the lived world. In other words, plans can become entrenched and acquire unduly high precision as I base my experience-dependent learning of policies on past perceptual inferences, yielding a “bad boot-strap” from past experience that renders me to unable to respond to a change in circumstances.

Bad boots-straps arise when an agent has prior beliefs that prevent them from learning adaptively about the environment, and are likely to result in inferences that seem optimal within a narrow frame of reference, but which are sub-optimal for the agent from a broader point of view. For instance, the agent may fail to have an accurate representation of the world, or try to optimize performance with respect to incomplete data; namely, data from the past that necessarily “ignores” data from the future. These ill-sampled actions or bad boot-straps show that it is not enough for an agent to bounce back to well-trodden paths, as those policies may be maladaptive; i.e., “I am stuck in a rut.” As there is no guarantee that the models entailed by agents are accurate representations of a changing reality, there is no guarantee that behavior premised on these beliefs will be optimal (Tschantz et al., 2020). For instance, the compulsion to consume potentially addictive stimulant drugs may be adaptive within a specific niche; e.g., the use of amphetamines or modafinil by soldiers on the battlefield. In such delimited situations, such actions may optimal (e.g., because they allow soldiers to remain awake and alert). From a broader point view, however, these actions can be detrimental, in the sense that lead to maladaptive states in the long term (e.g., leading to addiction after returning from service).

Mathematically, this phenomenon can be understood as getting trapped in *local minima*. If the agent’s beliefs about the world—or its engagement with that world—are too precise or restricted, then the agent denies itself the possibility of exploring alternative explanations or repertoires of behavior that would be more apt for a changed world (e.g., the world following the death of a loved one). What it would take to overcome these local minima is a broader vantage point from which the agent could see that there are alternative priors to explore. This might involve changing the structure of a generative model to include alternative hypotheses about ways of being. Alternatively, the requisite increase in the repertoire of priors follows from reducing the precision of high-level beliefs; enabling low-probability priors to compete on a more even footing. This is sometimes cast in terms of flattening the landscape of prior beliefs through reducing their precision (Hohwy et al., 2008; Carhart-Harris and Friston, 2019).

This idea has been formalized in active inference by means of generative models that are able to track both local and global precision dynamics (Miller et al., 2022). Local precision dynamics indicate our performance on specific tasks: e.g., in singing, being a good friend, at work, etc. We may optimize performance on each of these tasks specifically. Global precision dynamics indicate our overall performance, meaning how well we balance all these local aspects. If one fails to optimize global precision, one may only optimize locally. In such a case, epistemic actions will be useful to improve performance for a local task, but will fail to promote good overall performance. For example, one may imagine someone who puts all their energy into performing better and better at their job, while neglecting all other aspects of life, leading to depression. This would be an instance of high local precision on one specific aspect but low global precision dynamics due to the neglect of other aspects of life.

#### 4.2.2 Exploiting error dynamics and precision to find optimality

Optimality entails an optimal balance of epistemic and instrumental action. This balance depends upon the precision afforded prior preferences, relative to epistemic affordances; and the precision afforded free energy, relative to habitual priors. In short, a high-order kind of resilience—that rests on being able to revise the very fabric or structure of generative models—depends upon assigning the right precision or subpersonal confidence to various beliefs. It is perhaps interesting that the very systems that are thought to encode these kinds of precision are those that underwrite goal-directed behavior, responses to novelty and indeed, the very capacity to act: e.g., dopamine in the encoding of novelty and its role in Parkinson’s disease (Adams et al., 2013; Schwartenbeck et al., 2015, 2019). The implicit kind of resilience at this level of self-evidencing rests upon the ability to adapt by ensuring the right level of structure learning through not ascribing too much precision to prior beliefs. In short, this amounts to resilience as plasticity of a certain sort; namely, the ability to entertain new hypotheses and update the repertoire of explanations and plans that are evaluated in terms of variational and expected free energy. There are many examples of how this formulation translates into clinical practice; ranging from cognitive behavior therapy (CBT) that encourages the search for evidence against “bad bootstrap” priors, through to the relaxation of prior precision via psychedelic drugs that act on particular neuromodulatory (5-HT2A) receptors (Carhart-Harris and Friston, 2019).

In summary, resilience can arise from a system’s adaptive capabilities, wherein higher-level processes emerge from densely interacting components and processes unfolding at hierarchically lower levels, resulting in self-organization at multiple scales (Levin, 1998; Holling, 2001). The most important instrument for the development of resilience in this sense is the optimization of precision or learning rates (i.e., learnability or adaptability) that is inherent in many natural systems. Elastic resilience is the return to prior functioning (through homeostatic processes and selective sampling under active inference in general)—this is adaptive (or minimizes FE), but can get caught in local minima where elastic resilience prevents learning of larger scale state transitions, and where functioning at longer time scales is compromised (the world transitioned to another state for which the agent has no useful policy repertoire to return to low FE states). Hence, inertia and elasticity needs to be accompanied by plasticity.

### 4.3 Plasticity *via* redundancy and slope chasing

#### 4.3.1 Redundancy and degeneracy

Effective homeostasis and allostasis often require executing complex chains of actions, based on a model of possible situations and responses to them. These chains enable complex systems to infer pathways back to desired or preferred states after disturbances, and to plan ahead in ways that minimize future exposure to uncertainty. In order for systems to frequent a finite set of characteristic states despite variable circumstances, this model of possibilities and responses must be *redundant* to a certain degree: many states lead to a relatively small set of desirable outcomes. As discussed at length in Sajid et al. (2020)—and as we will unpack below—redundancy, although it increases the complexity (thus energy expense) of a model, is nonetheless crucial for resilience to the extent that it enables an agent to manage greater levels of uncertainty. This type of “useful redundancy” is often called *degeneracy*.

Whitacre and Bender (2010) define “degeneracy” in biological systems in a related way:

“Degeneracy is also known as partial redundancy. In biology it refers to conditions under which the functions or capabilities of components overlap partially (Edelman and Gally, 2001). It particularly describes the coexistence of structurally distinct components (genes, proteins, modules, or pathways) that can perform similar roles or are interchangeable under certain conditions, yet have distinct roles under other conditions.”

Degeneracy in this sense implies a many-to-one relationship among parts as discussed above, but also a one-to-many relationship, since partial redundancy requires that components have multiple functions (and thus variable relations to other parts). We note that this differs extensionally from the previous definition only in cases of pure redundancy, which arguably never occurs (e.g., the left kidney has the function of detoxifying the blood even if the right kidney is damaged, but the right kidney lacks this function).

As Sajid et al. (2020) argue, there is a simple relationship between degeneracy, in the sense of useful redundancy, and variational free energy, which—as discussed above—is the base quantity optimized in active inference (with expected free energy scoring the plausibility of a policy, given a temporally deep generative model that allows for planning based on expectations over future observations). The variational free energy (VFE) can be expressed in several ways, each of which illuminates a different aspect of the utility of VFE minimization. Degeneracy is most clearly evinced if the VFE is expressed as a Helmholtz free energy:


(1) $$F=\underset{E\,n\,e\,r\,g\,y}{\underbrace{{𝔼}_{Q}\,\left[-1\,n\,P\,\left(s,o\right)\right]}}-\underset{E\,n\,t\,r\,o\,p\,y}{\underbrace{{𝔼}_{Q}\,\left[-1\,n\,Q\,\left(s\right)\right]}}$$

The second term in (1) is simply the entropy of the recognition (approximate posterior) density *Q*(*s*), which (Sajid et al., 2020) argue to be formally equivalent to degeneracy. A second way of decomposing the VFE foregrounds the term 𝔼*_*Q*_*[ln *P* (*o*| *s*)], which measures the accuracy of using inferred hypotheses to predict sensory inputs (e.g., expected prediction error):


(2) $$F=\underset{C\,o\,m\,p\,l\,e\,x\,i\,t\,y}{\underbrace{D_{K\,L}\left[Q\left(s\right)||P\left(s\right)\right]}}-\underset{A\,c\,c\,u\,r\,a\,c\,y}{\underbrace{{𝔼}_{Q}\,\left[1\,n\,P\,\left(o|s\right)\right]}}$$

The first right-hand term is the KL divergence or asymmetrical difference between Q(s), the approximate posterior over hidden states, and P(s), the prior distribution over those same hidden states. This term is called complexity because it is a measure of “the degrees of freedom that are used to provide an accurate account of sensory data” (Sajid et al., 2020, p. 5,752). More simply, it scores the degree to which one changes one’s mind from beliefs prior to seeing some sensory evidence to the same beliefs *a posteriori*. This information gain is often considered as a complexity cost because the implicit erasure of information is energetically and metabolically expensive. In other words, assimilating some new sensory evidence entails a complexity cost that ensures belief updating provides an accurate account of sensations that is as simple as possible (i.e., does not diverge too much from prior beliefs), also thereby avoiding overfitting.

Given the definition of complexity, it is clear that more complex models are, *ipso facto*, less efficient: they explain a given sensory data-point using more degrees of freedom than alternative models. Complexity is then precisely the opposite of efficiency, which has been called redundancy in the literature: a redundant system is an inefficient one, since it uses the same resources that a simpler system would use to achieve the same goal, plus some extra resources (the redundancies).

Simply using log rules to expand the expression for complexity, i.e., the KL divergence from posterior to prior beliefs, we then obtain the following (cf. Sajid et al., 2020, p. 5,753):


(3) DK⁢L[Q(s)||P(s)]⏟C⁢o⁢m⁢p⁢l⁢e⁢x⁢i⁢t⁢y=𝔼Q⁢[-1⁢n⁢P⁢(S)]⏟cos′⁡t′-𝔼Q⁢[-1⁢n⁢Q⁢(s)]⏟D⁢e⁢g⁢e⁢n⁢e⁢r⁢a⁢c⁢y

This breakdown reveals a “residue” of complexity (“cost”) that is not exploited by the system in the service of sustaining a variety of useful means to an agent’s ends. Degeneracy, on the other hand, is the degree to which different causes in a system may lead to the same outcome, and the entropy of the recognition density (second right-hand term) quantifies precisely how many distinct states the agent believes to have some significant probability of producing a given outcome (observation).

Sajid et al. describe the “cost” in Eq. (3) as the negative expected “value” (log of prior preferences) of inferred states. This is one way of viewing why this term is “costly”: it quantifies the extent to which inferred states of affairs are a poor fit with an agent’s prior preferences (in this case, over states). But it is also possible to view this cost from an epistemic point of view. In epistemic terms, the residual complexity or “cost” term is a measure of how surprising the agent’s posterior inferences about the world are, on average, when measured against the agent’s own prior over hypotheses (specifically, it is the Shannon description length of the agent’s inferred states, under its generative distribution). Interestingly, in the case in which this cost term and the degeneracy are equal, the complexity vanishes—but the uncertainty remains. This suggests that the complexity term in effect encodes “useless uncertainty,” while the entropy over posterior beliefs (degeneracy) is useful or functional uncertainty. Intuitively, uncertainty is useful when it mirrors actual causes for uncertainty in the environment—that is, when the entropy of the belief distribution accurately models the entropy of the source of sensory signals. Given free energy as an objective function for inference and learning, we can expect the entropy of *Q* to be maximized, insofar as doing so does not impair accuracy (cf. Eq. 2 above). This is all entirely consistent with the basic physics of measurement (i.e., inference) and self-organization; ranging from Jaynes’ maximum entropy principle, through to universal computation via the minimization of algorithmic complexity and description lengths (Wallace and Dowe, 1999; Hutter, 2004; Sun et al., 2011; Ramstead et al., 2022).

The key take away is that, in order to minimize this type of functional, it is sufficient to change the prior belief distribution so that it matches the posterior distribution. If this is done optimally, then any remaining model complexity is due to the entropy of the posterior distribution itself. And for reasons that have been widely discussed (Jaynes, 1957; Sajid et al., 2020), it is optimal to maximize this entropy subject to constraints afforded by the generative model. Note here, that one can minimize variational free energy by changing priors; namely the model *per se*. This is the basis of structure learning that underwrites resilience as plasticity.

To return to the example of a soldier returning from war: addiction involves a highly precise (rigid) prior over specific states, which, among other problems, leads to acute suffering when the need for the addictive substance cannot be satisfied. Relaxing this prior preference distribution to match the wider range of states that is available once the solider returns home leverages model complexity in the service of degeneracy, trading rigidity for long-term resilience.

### 4.4 Slope chasing and error consumption

Having set up the temporally deep and hierarchically nested architecture of the generative models, we can now reconsider what optimal performance for such a system would look like. Here, we will argue that optimal performance occurs when the agent seeks out interesting slopes of error and has a propensity to actively seek out surprises. Technically speaking, this entails maximizing the epistemic part of expected free energy namely, maximizing expected information gain. Although it may sound paradoxical, this means that surprising events in the future are now attractive because they provide the opportunity to resolve uncertainty. This is reflected in the epistemic affordance that is often discussed in terms of saliency (resolving uncertainty about latent states) or novelty (resolving uncertainty about model parameters) (Schwartenbeck et al., 2019). As uncertainty is resolved, the precision of beliefs about states of the world—and action upon that world—changes. The dynamics of precision are thought by many to undergird affect and emotional valence (Joffily and Coricelli, 2013; Smith et al., 2019; Hesp et al., 2021).

Affective valence acts as a second-order form of information in the system, tuning our adaptive behaviors to changes in how our generation of error itself changes over time; specifically, changes relative to expectations about velocity and acceleration of error generation and minimization itself. Because of this, resilient systems must be slope chasers: they must always be on the lookout for optimal error reducing opportunities. We might call these optimal slopes “consumable errors”—errors that have the right amount of complexity so that the agent can learn at a good rate. Here, a good rate is one where the system is not overwhelmed by the situations that it encounters, while at the same time learning as much as possible about its environment, to perform better in forthcoming settings. These second-order, affective dynamics play a key role in keeping predictive agents poised, in a meta-stable fashion, between well-known and unknown niches—that is, at the edge of criticality, where they optimize learning rates and empowerment, exhibiting the right kind of resilient plasticity.

While systems consume error in order to be prepared for volatile onslaughts, some threshold of error will be beyond what a system can consume. In addition to the dangers outlined above concerning bad bootstraps, it is a fact that in uncertain environments, organisms do not succeed at managing errors for very long by retreating into well-known homeostatic (i.e., safe) situations and stereo- typed behaviors. In effect, some events will trigger cascades of surprising events, which even the most avoidant strategy cannot overcome. This emergence of this kind of error, by its very nature, is itself unpredictable, as it extends beyond the reach of the agent’s model. The best strategy, in this case, is to be a system which thrives in risky settings, quickly consumes errors thrown in its direction, and is able to grow from it.

## 5 Conclusion

Our aim in this article was to conceptualize—and formalize—the construct of resilience using the tools of active inference. When viewed through the lens of active inference and free energy minimization, this relational aspect becomes key: this follows from the fact that free energy scores the goodness of “fit” be- tween an agent and her physiological, physical or cultural niche. The existential imperatives—implicit in free energy minimization—are only defined in terms of how an agent actively engages with, or relates to, her environment.

We presented a conceptual analysis of resilience, distinguishing between meanings of the word “resilience”: (i) as inertia, or the capacity to resist change (ii) as elasticity, i.e., the capacity to bounce back from a perturbation, and (iii) as plasticity, the capacity to flexibly expand the repertoire of hypotheses and responses. We provided a formal interpretation of each aspect of the concepts via the active inference framework. In particular, we discussed resilience as inertia, which can be mapped onto high-precision beliefs about essential variables; resilience as elasticity, which ensures an ultimate return to characteristic states; and finally resilience as plasticity, which we unpack in terms of the learnability that underwrites functional redundancy and structural degeneracy. We hope these will provide first steps toward a formal (i.e., quantitative, computational) study of resilience. In follow up work, we plan to investigate the resilience of *communities*; notably by examining the concept of *sustainability*.

## Author contributions

MA and MM contributed to the conceptualization and writing of the manuscript. MR and KF have provided substantial edits and contributions to the core conceptualizations. RP, AK, JM, and CG have provided important contributions to the writing of this manuscript. All authors contributed to the article and approved the submitted version.

## Footnotes

1 Resilience is best understood in terms of degrees rather than a categorical trait. All agents can be considered resilient to some degree. However, those that are more resilient are such that they remain more stable in times of strong uncertainty and unexpected change in the environment. In light of this, this article intends to shine light on the qualities that allow an agent to with more stability to unexpected environmental change.
